# Imaging of pituitary tumors: an update with the 5th WHO Classifications—part 1. Pituitary neuroendocrine tumor (PitNET)/pituitary adenoma

**DOI:** 10.1007/s11604-023-01400-7

**Published:** 2023-02-24

**Authors:** Taro Tsukamoto, Yukio Miki

**Affiliations:** Department of Diagnostic and Interventional Radiology, Graduate School of Medicine, Osaka Metropolitan University, 1-4-3 Asahi-Machi, Abeno-Ku, Osaka, 545-8585 Japan

**Keywords:** Pituitary tumor, Pituitary adenoma, Pituitary neuroendocrine tumor (PitNET), WHO Classification of Tumors, MRI

## Abstract

The pituitary gland is the body’s master gland of the endocrine glands. Although it is a small organ, many types of tumors can develop within it. The recently revised fifth edition of the World Health Organization (WHO) classifications (2021 World Health Organization Classification of Central Nervous System Tumors and 2022 World Health Organization Classification of Endocrine and Neuroendocrine Tumors) revealed significant changes to the classification of pituitary adenomas, the most common type of pituitary gland tumor. This change categorized pituitary adenomas as neuroendocrine tumors and proposed the name to be revised to pituitary neuroendocrine tumor (PitNET). The International Classification of Diseases for Oncology behavior code for this tumor was previously “0” for benign tumor. In contrast, the fifth edition WHO classification has changed this code to “3” for primary malignant tumors as same to neuroendocrine tumor in other organs. Because the WHO classification made an important and significant change in the fundamental concept of the disease, in this paper, we will discuss the imaging diagnosis (magnetic resonance imaging, computed tomography, and positron emission tomography) of PitNET/pituitary adenoma in detail, considering these revisions as per the latest version of the WHO classification.

## Introduction

The pituitary gland is the master gland of the endocrine system and regulates the functions of the endocrine glands throughout the body. Although it is a small organ approximately the size of the tip of the little finger, both the anterior and posterior lobes are composed of a broad variety of cell types, and many types of tumors can develop within it. Imaging diagnosis techniques such as magnetic resonance imaging (MRI) play an important role in the diagnosis of tumors that develop in the pituitary gland. The recently revised fifth edition of the WHO classifications (2021 World Health Organization Classification of Central Nervous System Tumors and 2022 World Health Organization Classification of Endocrine and Neuroendocrine Tumors) has made significant changes to the classification of pituitary adenomas, the most common type of pituitary gland tumor (Table 1) [1, 2]. In this paper, we will discuss the imaging diagnosis (MRI, computed tomography [CT], positron emission tomography [PET]) of pituitary neuroendocrine tumor (PitNET)/pituitary adenoma in detail, considering these revisions to the latest version of the WHO classification.

**Table 1:** Key points in the 5th edition of the WHO classification of tumors of the pituitary region

• The most important point is the recommendation that pituitary adenoma be renamed as pituitary neuroendocrine tumor (PitNET). The International Classification of Diseases for Oncology (ICD-O) behavior code is revised from “0” to “3,” which indicates a change from benign to malignant disease. Pituitary carcinoma is also changed to metastatic PitNET
• Adamantinomatous craniopharyngioma and papillary craniopharyngioma are distinguished as separate tumor types
• Pituitary blastoma has been listed in the WHO Classification of Endocrine Tumors since the 4th edition and in the Central Nervous System WHO Classification since the 5th edition
• Pituicytoma, granular cell tumors of the sellar region, spindle cell oncocytoma, and sellar ependymoma are grouped into the pituicyte tumor family in the 5th edition of the WHO Classification of Endocrine Tumors
• Poorly differentiated chordoma *has been recognized as a subtype of chordoma with* clinicopathological features characterized by loss of SMARCB1 expression and is newly listed in the 5th edition of the WHO Classification of Endocrine and Neuroendocrine Tumors

## Pituitary neuroendocrine tumor (PitNET)/pituitary adenoma

### Definition and epidemiology

PitNET/pituitary adenoma is defined as a clonal neoplastic proliferation of the anterior pituitary hormone-producing cells [2]. They occur with a mean incidence of approximately 5.1 cases per 100,000 annually [3]. PitNET/pituitary adenoma accounts for approximately 15% of all primary brain tumors, making it the third most common tumor after meningiomas and diffuse astrocytic and oligodendroglial tumors [4]. Based on the autopsy study, the estimated prevalence is 16.7%, including incidental PitNET/pituitary adenomas [5]. The sex ratio is slightly higher in females, and it is rare in children. In children, the ratio is 1.8:1 with a female preponderance [6]. The frequency of types is as follows: non-functional is at 57% and functional is at 43% (growth hormone [GH]-producing, 18%; prolactin [PRL]-producing, 12%; adrenocorticotrophic hormone [ACTH]-producing, 5%; gonadotropin-producing, 5%; GH-PRL-producing, 1%; and thyroid-stimulating hormone [TSH]-producing, 1%) [7].

### New (5th edition) WHO Classifications

#### Name change

**Fig. 1:**
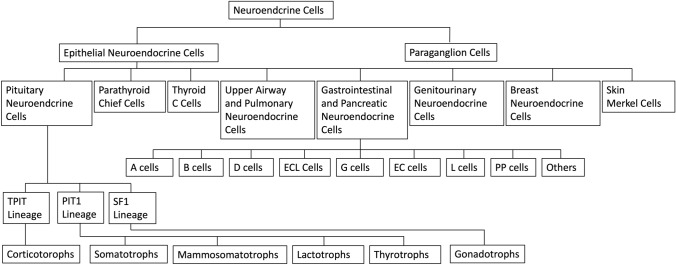
The families of neuroendocrine cells. (Reproduced with permission from Springer Nature [11])

The term “pituitary adenoma” has been used since 1932 when Harvey Cushing first proposed this term [8]. This tumor has since been treated as a benign tumor. However, it has several characteristics that distinguish it from ordinary benign tumors, including a tendency for hemorrhage and necrosis, frequent invasion of nearby structures, poor patient prognosis, and rare metastasis. The tumor also expresses neuroendocrine proteins such as synaptophysin, chromogranin A, CD56, and insulinoma-like protein 1, which are characteristics of neuroendocrine tumors [9]. A common classification framework has been proposed for neuroendocrine tumors, rather than organ-specific classification [10]. Therefore, pituitary adenomas are now being classified as neuroendocrine tumors rather than adenomas (Fig. 1) [11]. First, the International Pituitary Pathology Club proposed changing the name of the disease [12]. Subsequently, the WHO/International Agency for Research on Cancer consensus proposal aimed to unify the approach to neuroendocrine tumors [10]. In response, in the WHO Classification of Central Nervous System Tumors 5th edition released in 2021, “pituitary adenoma” was incorporated under the same entry as “PitNET”, appearing as “pituitary adenoma/PitNET.” In the 4th edition of the WHO classification, some pituitary tumors, including pituitary adenomas, were listed only in the WHO Classification of Endocrine Tumors. However, in the 5th edition, it is listed in both the WHO Classification of Endocrine and Neuroendocrine Tumors and the WHO Classification of Brain Tumors (Table 2) [1, 2]. In the WHO Classification of Endocrine and Neuroendocrine Tumors 5th edition released in 2022, it is listed as “PitNET/pituitary adenoma” and recommends the use of PitNET instead of “pituitary adenoma.” Tumor definitions are listed by type, for example, “somatotroph PitNET/pituitary adenoma” is described as “A well-differentiated pituitary/neuroendocrine tumor composed of PIT1-lineage adenohypophysial cells with somatotroph differentiation.” The definition now includes pituitary neuroendocrine tumor, transcription (e.g., PIT1), and the name of the cell from which it differentiated (e.g., adenohypophysial cells with somatotroph differentiation). In the 5th edition, the behavior code for International Classification of Diseases (ICD) coding was revised from “0” for benign tumors to “3” for primary malignant tumors as same to neuroendocrine tumors in other organs [1, 2].

**Table 2:** Pituitary neuroendocrine tumor (PitNET)/pituitary adenoma as described by WHO classification of 4th and 5th edition

	Tumors of the central nervous system	Central nervous system tumors
Edition	4th	5th
Issue year	2016	2021
Terminology	Not listed	Pituitary adenoma/Pituitary neuroendocrine tumor (PitNET)
Definition		Clonal neoplastic proliferation of anterior pituitary hormone-producing cell
ICD-O coding		8272/3 (3: primary malignant tumor)

	Tumor of endocrine organs	Endocrine and neuroendocrine tumors
Edition	4th	5th
Issue year	2017	2022
Terminology	Pituitary adenoma	Pituitary neuroendocrine tumor (PitNET)
Definition	Neoplastic proliferation of anterior pituitary hormone-producing cells	Defined for each type
ICD-O coding	8272/0 (0: benign tumor)	8272/3 (3: primary malignant tumor)

As the term “adenoma” originally referred to tumors derived from epithelial cells and tumors arising from cells with secretory granules should be classified as neuroendocrine tumors, the name change in the 5th edition of the WHO classification seems appropriate. However, treating frequently occurring tumors that have been considered benign as malignant tumors may impose a greater psychological burden than necessary by informing patients who have no symptoms of incidentally discovered tumors that they may have malignant tumors and may cause various social and medical economic problems. Considerable time will likely be necessary for the change in the disease name to gain social consensus and awareness [13, 14]. Pituitary adenomas not only involve the field of neurosurgery and endocrinology, but also ophthalmology, obstetrics and gynecology, otolaryngology, and many other medical departments. Until the name PitNET is widely accepted by most physicians in most departments, it may be advisable to include the name pituitary adenoma along with the new name PitNET in the radiologist’s imaging report.

#### Pathologic classification

**Fig. 2:**
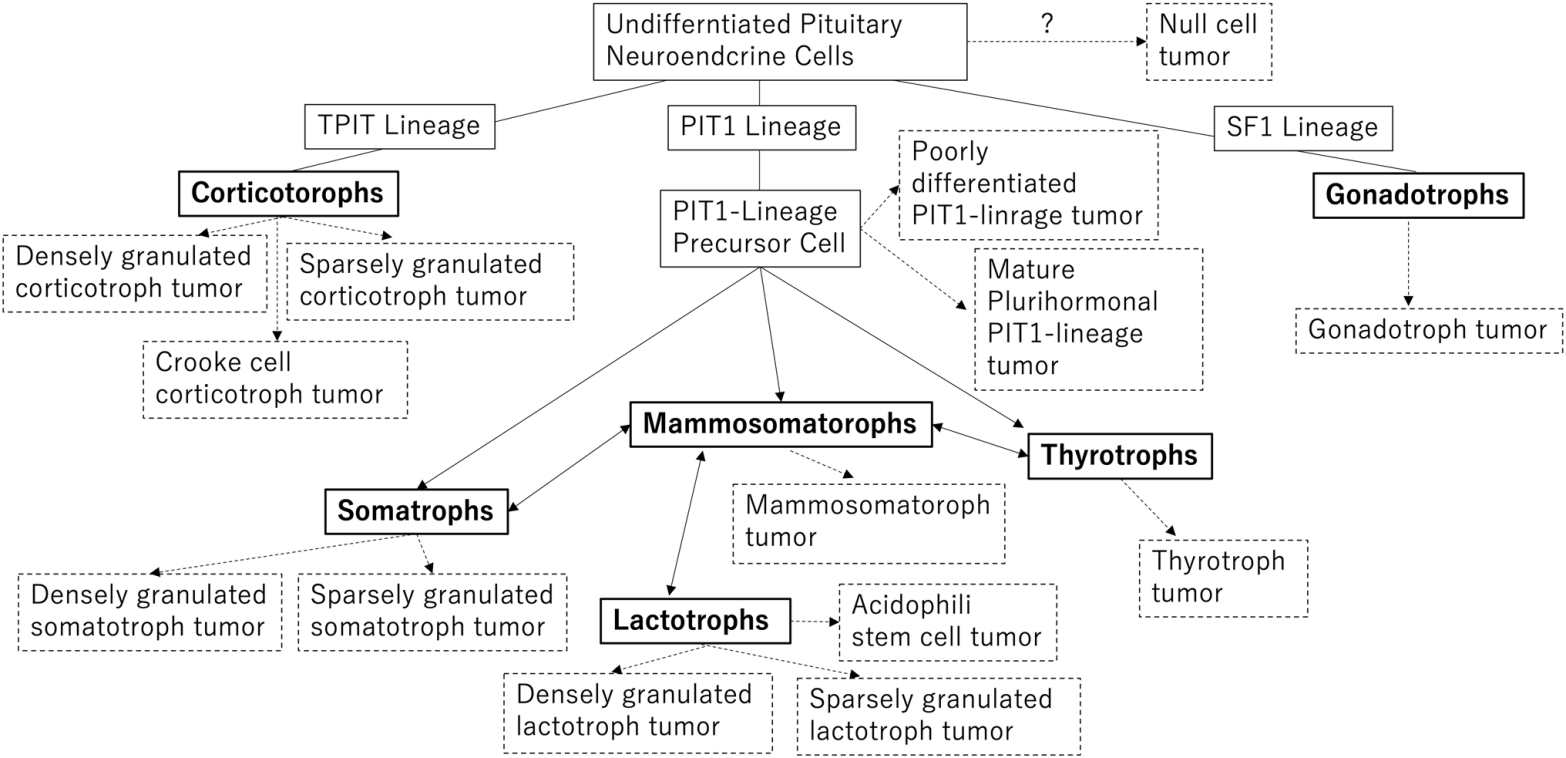
Pituitary cell differentiation and PitNETs. Thick lines are normal pituitary cells; dashed lines are PitNETs. (Reproduced with permission from Springer Nature [11])

The new WHO classification of PitNET/pituitary adenoma is shown in Table 3 [15]. Since the WHO Classification of Endocrine Tumors 4th edition, the histological type is now defined not only by the anterior pituitary hormones (GH, PRL, ACTH, TSH, follicular and luteinizing hormones), but also by the transcription factors involved in the differentiation of anterior pituitary cells. The new 5th edition of the WHO classification also retains the transcription factor-based histology. The relationship between the anterior pituitary cell lineage and transcription factors is shown in Fig. 2. The anterior pituitary hormone-secreting cells are divided into three major groups (PIT1, TPIT, and SF1) according to the transcription factors. There are somatotrophs, lactotrophs, mammosomatotrophs, and thyrotrophs in the PIT1 group; corticotrophs in the TPIT group; and somatotrophs in the SF1 group. Anterior pituitary hormone-secreting cells can physiologically differentiate between the PIT1 group and other cell types [16]. PitNET/pituitary adenoma, the PIT1 group in the new WHO classification, includes somatotroph tumors, lactotroph tumors, mammosomatotroph tumors, thyrotroph tumors, other mature plurihormonal PIT1-lineage tumors, immature PIT1-lineage tumors, acidophil stem cell tumors, and mixed somatotroph and lactotroph tumors. Corticotroph tumors are included in the TPIT group and somatotroph tumors in the SF1 group. Mammosomatotroph tumors and acidophil stem cell tumors are classified as a type of PIT1 group tumor in the new WHO classification. Plurihormonal PIT-1-positive adenoma, introduced in the 4th edition of the WHO classification, was divided into two categories in the 5th edition of the WHO classification: immature PIT1-lineage tumor (formerly silent subtype 3 adenoma) and mature plurihormonal PIT1-lineage tumors.

**Table 3:** Types and subtypes of PitNET [15]

	PitNET type	Subtype
PIT1-lineage	Somatotroph tumors	Densely granulated somatotroph tumor
		Sparsely granulated somatotroph tumor
	Lactotroph tumors	Sparsely granulated lactotroph tumor
		Densely granulated lactotroph tumor
	Mammosomatotroph tumor	
	Thyrotroph tumor	
	Mature plurihormonal PIT1-lineage tumor	
	Immature PIT1-lineage tumor	
	Acidophil stem cell tumor	
	Mixed somatotroph and lactotroph tumor	
TPIT-lineage	Corticotroph tumors	Densely granulated corticotroph tumor
		Sparsely granulated corticotroph tumor
		Crooke cell tumor
SF1-lineage	Gonadotroph tumor	
No distinct cell lineage	Plurihormonal tumor	
	Null cell tumor

Somatotroph tumors, lactotroph tumors, and corticotroph tumors are divided into two subtypes: densely granulated with abundant intracytoplasmic secretory granules and sparsely granulated with poor intracytoplasmic secretory granules. Although electron microscopy was used for differentiation in the past, evaluation using electron microscopy are not required. Corticotroph tumors also have a subtype in which the tumor cells exhibit Crooke degeneration.

Although null cell tumors were previously defined as tumors that were hormone negative, since the 4th edition of the WHO classification, they have been defined by their lack of transcription factors (PIT1, TPIT, SF1, and GATA3). Tumors that are hormone negative account for approximately 20–30% of nonfunctioning tumors, while null cell tumors are very rare, accounting for < 5% [17]. Some argue that null cell tumors may not actually exist [4].

Although “atypical adenoma” was defined by the WHO in 2004 as “increased fission, Ki-67 labeling index > 3%, and positive p53 immunostaining, not all tumors that meet the criteria show an aggressive course, and even ordinary pituitary adenomas may meet the criteria; thus, “atypical adenoma” was deleted in the 4th edition of the WHO classification [17].

The nuclear fission number, Ki-67, and p53 expression are controversial markers for determining the aggressive pattern of PitNET/pituitary adenoma, and many other markers have been proposed, but all are currently under investigation [18, 19]. Meanwhile, the course of the disease is known to differ by histological type. Gonadotroph tumors are known to exhibit indolent behavior, especially in elderly patients. Somatotroph tumors, densely granulated lactotroph tumors, acidophil stem cell tumors, sparsely granulated corticotroph tumors, Crooke’s cell tumors, immature PIT1-lineage tumors, null cell tumors, and others have been said to exhibit an aggressive course [20].

#### Metastatic PitNET

Metastatic PitNETs are PitNETs that have metastasized to lymph nodes and distant sites or have demonstrated discontinuous spread through the central nervous system. In the WHO classification up to the 4th edition, pituitary adenomas that had not metastasized were considered benign, while pituitary adenomas that had metastasized were called “pituitary carcinomas” and were classified as malignant. The latest 5th edition of the WHO classification classifies PitNETs as malignant tumors; thus, metastatic tumors are now called “metastatic PitNETs” instead of “pituitary carcinomas.” Metastatic lesions are not poorly differentiated and cannot be pathologically distinguished from typical PitNET/pituitary adenomas [2]. Metastatic PitNETs are rare tumors constituting only 0.12% of pituitary tumors and 0.4 and 0.56% of PitNETs [21]. Most cases present as invasive PitNETs/pituitary adenomas and eventually metastasize. The average survival is < 4 years [22]. The most common tumor type is the lactotroph tumor or corticotroph tumor [23].

### Hereditary syndromes

PitNET/pituitary adenoma may occur in association with some very rare genetic syndromes. The majority of cases involves multiple endocrine neoplasia type 1 (MEN 1), with approximately half of MEN 1 cases having PitNET/pituitary adenoma, and approximately 10% having first symptoms of PitNET/pituitary adenoma [24]. Other known rare pathologies include Carney complex, familial isolated pituitary adenoma [25], isolated familial somatotropinoma [26], and X-linked acrogigantism [27].

### Symptoms

Symptoms of PitNET/pituitary adenoma may be due to the hormones produced by the tumor or to pressure drainage to nearby structures.

Acromegaly occurs in GH hormone-producing tumors, 98% of which is caused by PitNET/pituitary adenoma [28]. Acromegaly is present when it occurs after the closure of the epiphyseal line, but if it occurs before the closure of the epiphyseal line, the patient develops gigantism. Symptoms of acromegaly include enlarged limbs, changes in facial appearance (brow arch bulging, enlarged nose and lips, protruding mandible, and giant tongue), hyperhidrosis, abnormal menstruation, sleep apnea, abnormal glucose tolerance, hypertension, malocclusion, carpal tunnel syndrome, and osteoarthritis [29].

In the case of PRL-producing tumors, hyperprolactinemia occurs. This pathology causes menstrual irregularities and amenorrhea, infertility, and lactation in women, as well as diminished libido, impotence, and gynecomastia in men. Approximately 50% of women and 35% of men experience milk secretion[29]. Bone loss occurs secondary to sex steroid depletion via hyperprolactinemia [30].

In the case of ACTH-producing tumors, hypercortisolemia results in Cushing’s syndrome. Fatigue, weight gain, swelling, menstrual abnormalities, hypertrichosis, acne, bruising, hyperpigmentation, depression, abnormal glucose tolerance, hypertension, and lipid abnormalities are common [29].

In TSH-producing tumors, blood thyroid hormone levels are elevated, and palpitations, hand tremors, excessive sweating, and weight loss may occur [29].

More than 99% of gonadotropin-producing tumors is asymptomatic [31]. In symptomatic cases, women have been reported to have developed menstrual irregularity, abdominal distension or increasing abdominal girth, amenorrhea, galactorrhea, abdominal or pelvic pain, hypomenorrhea, hypermenorrhea, infertility, or ovarian hyperstimulation, while men develop enlarged testes [32].

Non-functioning tumors include “silent pituitary PitNETs/pituitary adenomas” that either do not produce hormones or do produce hormones but do not have clinical hormone-related symptoms. Histology is often positive for gonadotropins. The tumor compresses the optic nerve, causing visual acuity and visual field deficits. Compression of the normal pituitary gland can cause hypopituitarism, although central diabetes insipidus (arginine vasopressin deficiency) is rare. Hyperprolactinemia (25–65%) is caused by the compression of the pituitary stalk (stalk effect) [33]. This is due to the suppression of dopamine activity, which is a PRL inhibitor.

### Imaging diagnosis

#### Routine MRI

MRI is the most useful imaging modality of choice in the diagnosis of PitNET/pituitary adenoma. The popular MRI sequences are spin-echo (SE) non-contrast T1-weighted sagittal and coronal sections, fast SE T2-weighted coronal sections, and contrast-enhanced T1-weighted coronal sections. For simple screening examinations, only non-contrast MRI is performed and contrast-enhanced MRI may not be performed. Axial T2-weighted or fluid-attenuated inversion recovery imaging of the whole brain is also recommended to exclude incidental or concurrent brain lesions.

MRI of the pituitary gland requires some ingenuity because of its small size, its proximity to bone and sinusoidal air, and the proximity of the internal carotid artery. To obtain images with high spatial resolution, slice thickness should be ≤ 3 mm, and the fine matrix (256 × 256 or more) setting with a small field of view (≤ 20 cm) should be used [34]. As susceptibility artifacts are relatively large due to the influence of bone and air, gradient-echo imaging is not suitable for this region, and imaging using the SE or fast SE method is generally used. To observe the posterior pituitary high signal, in T1-weighted sagittal sections, the frequency-encoding direction should be shifted back and forth so that the chemical shift artifact in the nearby marrow fat does not overlap with the pituitary gland [35], or fat suppression should be used. In coronal sections, the phase-encoding direction is inferosuperiorly so that flow artifacts in the internal carotid artery do not overlap with the pituitary gland; 1.5 T MRI can usually detect the lesion; but 3 T MRI is more sensitive for microadenoma. It is also easier to distinguish PitNETs/pituitary adenomas from surrounding structures [36, 37].

Dynamic MRI is useful for visualizing microadenoma and the normal pituitary gland compressed by a macroadenoma [38]. Dynamic MRI involves rapid (≥ 4 mL/s) intravenous injection of gadolinium-based contrast media and repeated imaging for a short period [34]. Dynamic MRI is usually performed with a fast SE coronal section, but if the microadenoma is located at the anterior or posterior end of the anterior lobe, the partial volume effect may make the diagnosis difficult. In such cases, three-dimensional (3D) dynamic MRI [39–42] or dynamic MRI with simultaneous acquisition of coronal and sagittal images [43] may be useful.

### Advanced MRI

#### Diffusion-weighted images (DWI)

Many attempts at DWI of PitNET/pituitary adenoma have been reported. The evaluation of pituitary lesions by single-shot echo planar imaging, a common method of diffusion-weighted imaging, is limited to macroadenomas and pituitary abscesses due to susceptibility artifacts. Yiping et al. [44] reported that PROPELLER/BLADE can enhance the image quality. Hiwatashi et al. [45, 46] suggested that diffusion-weighted images using 3D turbo field-echo with diffusion-sensitized driven-equilibrium preparation are suitable for the evaluation of the pituitary gland and PitNETs/pituitary adenomas. Wang et al. [47] suggested that field of view optimized and constrained undistorted single-shot (FOCUS) DWI can obtain high-resolution images of the pituitary region in a clinically feasible scan time (1 min 30 s). Intravoxel incoherent motion (IVIM) of the anterior pituitary has been reported to be evaluated with turbo SE DWI [48]. The perfusion fraction measured by IVIM may be useful in the pituitary region as a new parameter [48]. In addition, comparison of apparent diffusion coefficient (ADC) values between nonfunctioning PitNETs/pituitary adenomas and sellar meningiomas has been reported to be significantly higher in nonfunctioning PitNETs/pituitary adenomas [49].

#### Arterial spin labeling (ASL)

Several reports regarding ASL for PitNETs/pituitary adenomas exist. The blood flow of nonfunctioning pituitary macroadenomas measured by ASL perfusion imaging reflects pathologic vascular density, and it may be useful for preoperative prediction of intraoperative or postoperative tumor hemorrhage [50]. Tumor blood flow (TBF) in GH-producing PitNETs/pituitary adenomas measured by ASL is reported to decrease after octreotide treatment, reflecting the antiangiogenic effect of octreotide [51]. TBF measured by ASL has also been reported to be significantly higher in suprasellar meningiomas (absolute median TBF; 172.95 mL/100 g/min) than in PitNETs/pituitary adenomas (absolute median TBF; 34.57 mL/100 g/min) [52].

#### Imaging to predict tumor stiffness

Because 5–13% of PitNETs/pituitary adenomas are stiff, which can make transsphenoidal endoscopic surgery difficult, it is desirable to be able to estimate tumor stiffness preoperatively. Although several studies have assessed the stiffness of the tumor using T2-weighted image (T2WI) or DWI, the stiffness of PitNETs/pituitary adenomas remains unclear [53–59]. With other ways such as the T2WI texture analysis and machine learning [60], MR elastography [61–63], dynamic MRI [64], and MR textural analysis on contrast-enhanced 3D-SPACE images [65], several studies have been reported to assess the stiffness of the PitNETs/pituitary adenomas.

### MRI findings

#### General characteristics of PitNET/pituitary adenoma on MRI

The signal intensity on MRI varies from case to case because components such as water are not constant between PitNET/pituitary adenoma, and modifications such as degeneration, hemorrhage, and infarction also develop. T1-weighted image (T1WI) often shows a mildly hypointensity compared to the normal pituitary, but may be isoitense, while T2WI may show a variety of signal intensities from low to high compared to the normal pituitary gland. On contrast-enhanced MRI, PitNETs/pituitary adenomas are often mildly hypointensity compared to the normal pituitary glands but are often iso-intensity as well. Imaging strategies for MRI vary depending on the size of the PitNET/pituitary adenoma and the hormones it produces.

## Strategies for imaging diagnosis based on size

### Microadenoma

Main purpose of imaging diagnosis is to determine the presence and localization of the PitNET/pituitary adenoma. For this purpose, it is necessary to contrast the PitNET/pituitary adenoma with the normal pituitary gland as much as possible.

**Fig. 3:**
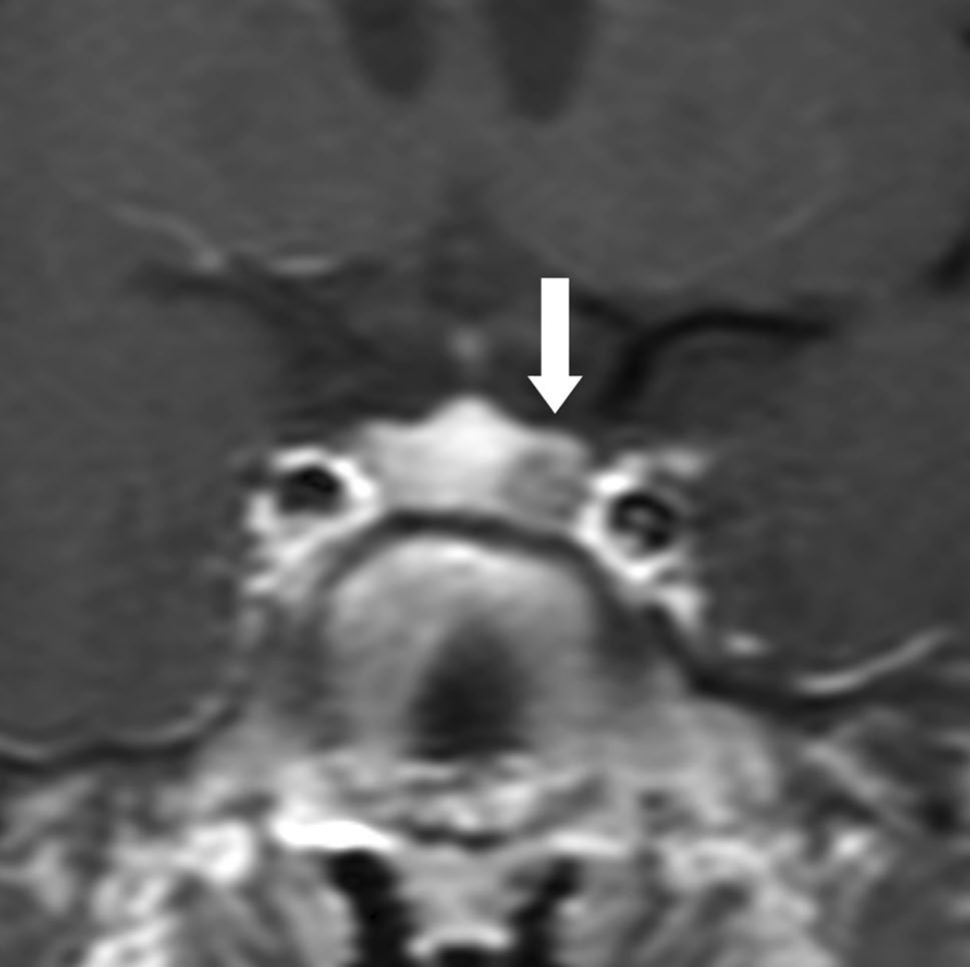
Microadenoma (Adrenocorticotrophic hormone [ACTH]-producing PitNET/pituitary adenoma). A 14-year-old male, with moon face and ACTH level of 91 pg/mL. Coronal dynamic T1WI (at 90 s) revealed a 5-mm mass clearly visible on the left side of the pituitary gland (arrow). A transsphenoidal approach was used to remove the tumor, which was confirmed to be an ACTH-producing adenoma

Dynamic MRI is most useful in localizing microadenomas [38, 66, 67]. Many PitNETs/pituitary adenomas have a later peak of contrast than the normal pituitary, with the contrast between the two being most pronounced at 1–2 min (Fig. 3) [38]. With dynamic MRI, attention to changes over time in signal intensity at each site due to contrast, rather than simply going for areas of weak contrast enhancement in the early phase after contrast administration, can reduce false-positive findings that misdiagnose magnetic susceptibility artifacts/air and bone partial volume effects as PitNETs/pituitary adenomas. In addition, if dynamic MRI is not performed and a normal contrast image is taken immediately after contrast administration, the limbus of the normal anterior lobe may show false-positive findings due to delayed arrival of the contrast agent [68]. However, such false-positive findings can be avoided with dynamic MRI.

Microadenomas may be microscopic in size, multiple, or have indistinct borders [69], and as a result, may not be detected. As such, high spatial resolution is needed to diagnose small microadenomas. The use of 1–1.2-mm-thin slice thickness in post-contrast spoiled gradient-echo 3D T1-weighted sequences (e.g., VIBE) has been reported to increase the detection sensitivity of microadenomas [70, 71]. Thin-slice pituitary MRI with deep learning-based reconstruction [72] may be useful in the diagnosis of microadenomas.

### Macroadenoma

PitNETs/pituitary adenomas larger than 1 cm in diameter are called macroadenomas. In cases involving macroadenomas, in addition to diagnosing the presence of a tumor, the goal of diagnostic imaging is to determine the internal characteristics (cystic degeneration, hemorrhage, etc.), morphology, and extent of the tumor; to identify the location of the normal pituitary gland; and to determine the presence or absence and location of compression to the optic chiasm and optic nerve; the presence or absence and condition of the cavernous sinus extension; the presence or absence and condition of osteoclastic activity; and the location of the main trunk artery.

Identifying the location of the normal pituitary gland in macroadenomas is an important preoperative information [68], and dynamic MRI can show a high percentage of the normal pituitary gland with an earlier contrast peak than in PitNETs/pituitary adenomas and a high percentage of the normal pituitary gland with compression [38].

**Fig. 4:**
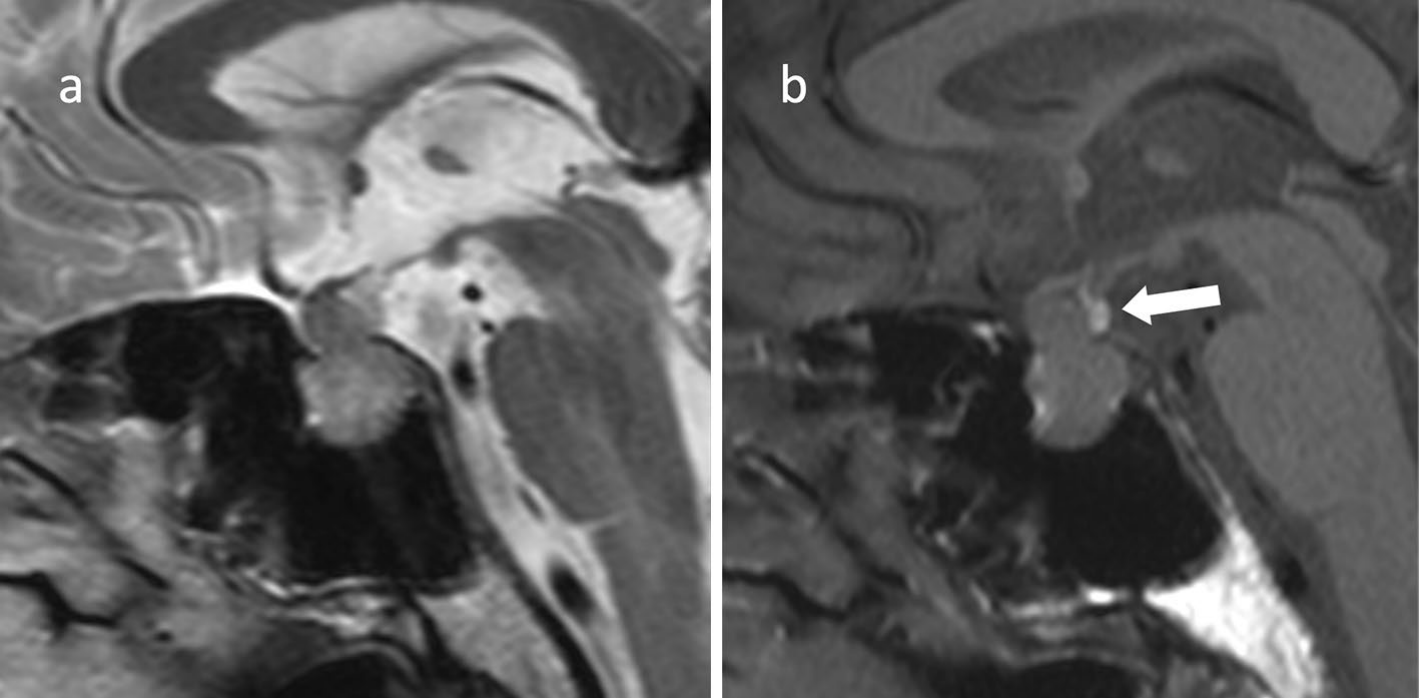
Non-functioning PitNET/pituitary adenoma. A male patient in his 60 s, with generalized body malaise. **a** Sagittal T2WI revealed a mass within the sella turcica to the suprasellar region. The mass is necked by the sella diaphragm and has a snowball shape. **b** Sagittal T1WI indicated a hyperintensity area in the posterior superior part of the mass (arrow), believed to be an ectopic posterior lobe

When extending to the suprasellar region, it is often constricted by the sella diaphragm, giving it a snowman shape (Fig. 4) [68]. If the optic chiasm is compressed, it is usually compressed superiorly to posteriorly, but if the optic chiasm is compressed anteriorly to the tumor (prefixed chiasm), surgical manipulation may be difficult and is an important preoperative information. The heavily T2WI, CE-FIESTA, has been reported to be useful in observing the optic chiasm and optic nerve compressed by the PitNET/pituitary adenoma [73, 74]. Hyperintensity of the optic nerve on T2WI due to compression of the mass is associated with visual impairment [75].

**Fig. 5:**
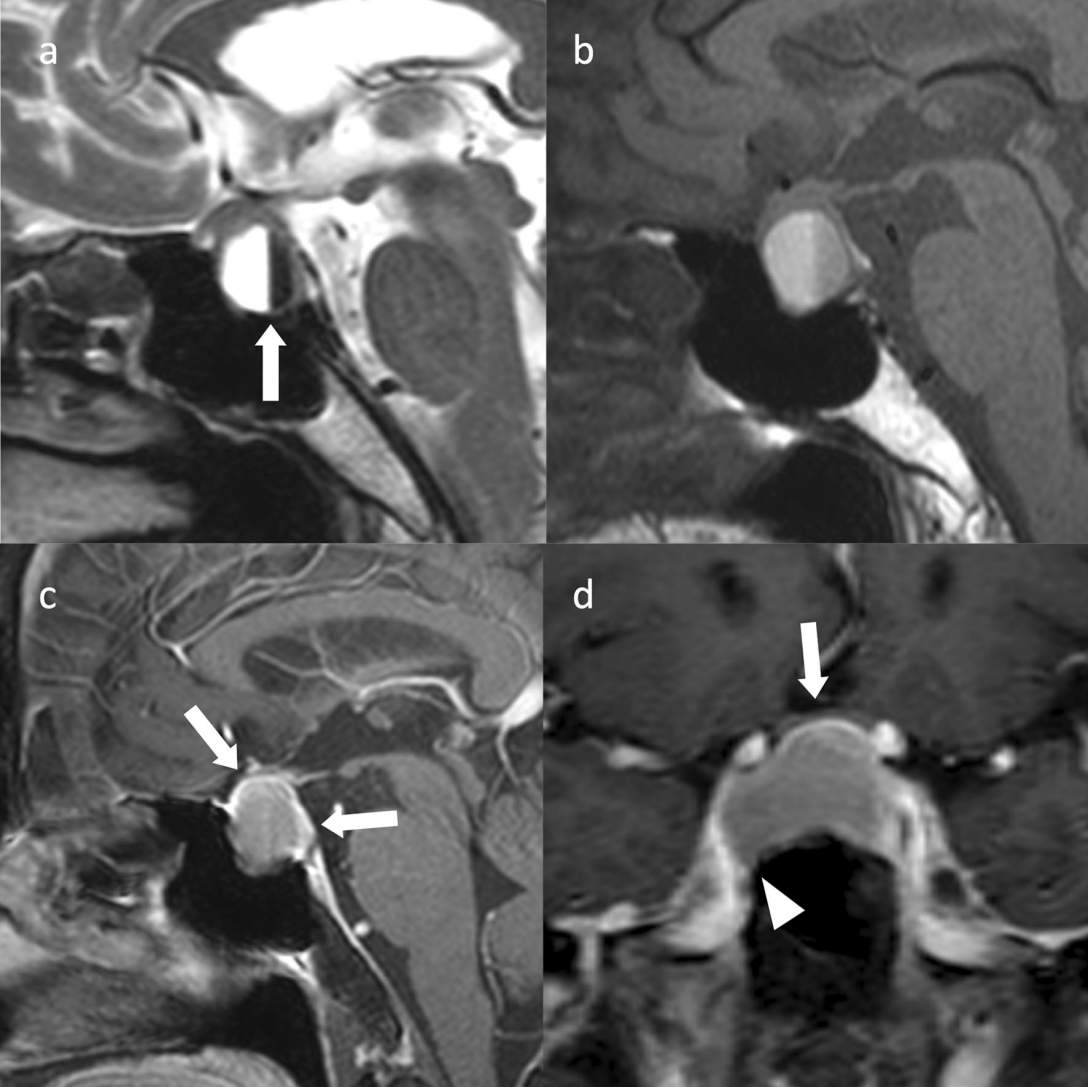
Cystic nonfunctioning PitNET/pituitary adenoma with internal bleeding. A female patient in her 70 s with bilateral auricular hemianopsia. A cystic mass is observed within the sella turcica to the suprasellar region. **a** Sagittal T2WI revealed a fluid–fluid level (arrow) with markedly hyperintensity anteriorly and markedly hypointensity posteriorly. **b** Sagittal T1WI shows hyperintensity anteriorly and mildly hypointensity posteriorly. **c** Sagittal contrast-enhanced T1WI shows a solid area of contrast at the limbus (arrow), which is believed to be a tumor component or pituitary gland. **d** The optic chiasm is slightly compressed upward by the mass on coronal contrast-enhanced T1WI (arrow). The mass protrudes into the right cavernous sinus and is classified as Knops grade 2 (arrowhead). The patient underwent surgery via the transsphenoidal sinus technique and diagnosed as a nonfunctioning PitNET/pituitary adenoma. No invasion of the cavernous sinus was observed

Macroadenomas often present as cystic masses. Approximately half of them has a fluid–fluid level within the cyst, reflecting hemorrhage (Fig. 5) [76]. Fluid–fluid levels are rarely seen in Rathke’s cleft cysts or adamantinomatous craniopharyngiomas. When a fluid–fluid level is seen in a cystic mass in the sellar region, it is often a cystic PitNET/pituitary adenoma [77].

Evaluation of cavernous sinus involvement provides critical preoperative information. The Knops classification is often used to evaluate lateral extension (Fig. 5d), with 1.5% of patients having grade 1, 9.9% grade 2, 37.9% grade 3, and 100% grade 4 involvement of the cavernous sinus [78]. Micko et al. [78] recommended dividing grade 3 into 3A (26.5% invasion into the cavernous sinus), which extends into the compartment above the internal carotid artery in the cavernous sinus, and 3B (70.6% invasion into the cavernous sinus), which extends into the inferior compartment. However, the Knops classification and the modified Knops classification by Micko et al. are less reliable for intermediate grades, and it may be difficult to distinguish compression from invasion of the cavernous sinus [79]. High-resolution T2-weighted or proton density-weighted images on 3 T MRI can show disruption of the medial wall of the cavernous sinus and may reveal cavernous sinus extension [68, 80]. Contrast-enhanced multi-detector CT may provide a more detailed picture of cavernous sinus extension than MRI [81]. Deep-learning reconstruction of 1-mm thin slices after contrast-enhanced MRI has also been reported to be highly diagnostic in identifying cavernous sinus invasion [72].

## MRI diagnostic strategies by hormone production

### Somatotroph PitNET/pituitary adenoma (GH-producing PitNET/pituitary adenoma)

**Fig. 6:**
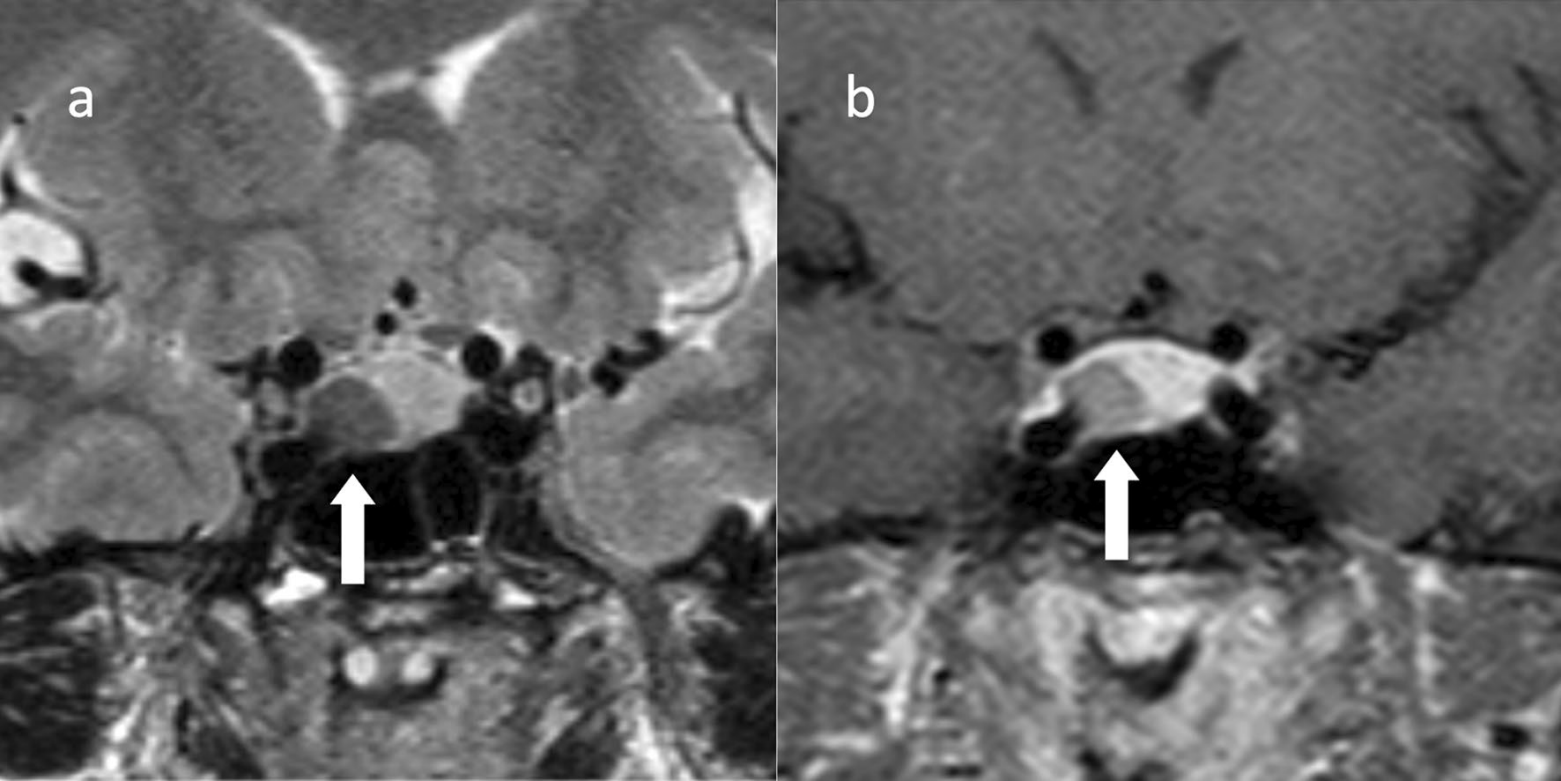
Growth hormone (GH)-producing PitNET/pituitary adenoma (suspected to be a densely granulated tumor). A 51-year-old female, whose shoe size and ring no longer fit. The GH level is at 13.9 ng/mL and the IGF-1 level is at 589 ng/mL. **a** Coronal T2WI shows hypointensity mass (arrow). **b** Coronal contrast-enhanced T1WI shows a hypointensity mass (arrow). The patient underwent surgery via the transiliac sinus approach. Pathologically, the patient was diagnosed as a GH-producing pituitary PitNET/pituitary adenoma. No medical treatment was administered. GH and IGF-1 levels subsequently normalized

T1WI often shows equal or slightly hypointensity, and T2WI often shows hypointensity (Fig. 6). The hypointensity compared to the gray matter on T2WI is considered to be characteristic of densely granulated tumors [82]. Densely granulated tumors often have a weaker contrast enhancement than sparsely granulated tumors [83]. In a report examining the signal enhancement rate of PitNETs/pituitary adenomas relative to normal brain tissue (putamen) during the early (within 39 s) and delayed (195 s) phases of dynamic MRI, GH-producing tumors had lower contrast enhancement rates than other PitNETs/pituitary adenomas in both the early and delayed phases, suggesting that the signal enhancement rate on dynamic MRI may be a useful parameter for distinguishing GH-producing PitNETs/pituitary adenomas from other PitNETs/pituitary adenomas [84]. It has also been suggested that the signal enhancement curve of the tumor on dynamic MRI may be useful for diagnosis [85, 86]. Sparsely granulated tumors are larger than densely granulated tumors and tend to be more invasive [87]. In addition, sparsely granulated tumors often destroy the sella turcica and extend inferiorly, and there are often cases in which sparsely granulated tumors do not form a mass within the sella turcica but only extend inferiorly [81, 82]. The reason for the downward extension may be due to bone fragility caused by excessive GH. In contrast, upward extension is less frequent, and therefore, visual field disturbance is less frequent [68].

Although surgery is the first choice of treatment, pharmacotherapy with somatostatin receptor ligands may be administered before or after surgery [88]. First-generation somatostatin receptor ligands act on the somatostatin receptor subtype 2 (SSTR2) and are considered to have a greater effect on densely granulated tumors [88, 89]. Pasireotide long-acting release, which acts on the somatostatin receptor subtype 5 (SSTR5), is used when the tumor is resistant to first-generation drugs. However, it has been reported that the response may be greater in sparsely granulated tumors [90].

### Lactotroph PitNET/pituitary adenoma (prolactinoma)

There is a relationship between serum PRL levels and the presence or absence and size of tumors (Table 4). PRL levels greater than 200 ng/mL indicate a PRL-producing PitNET/pituitary adenoma, often a macroadenoma (macroprolactinoma) larger than 1 cm in size [68]. A PRL-producing tumor with a level greater than 1000 ng/mL suggests a macroprolactinoma with cavernous sinus extension [69].

**Table 4:** Relationship between serum prolactin levels and causes [68, 69]

Prolactin levels	Causes
< 100 ng/mL	• Stalk effect (increased prolactin due to impaired prolactin inhibitory factor delivery by compression to the pituitary stalk): nonfunctioning pituitary macroadenoma, Rathke’ cleft cyst, craniopharyngioma, germinoma, etc.
	• Drugs: Anti-ulcer drug, antiemetic drug, antihypertensive drug, psychotropic drug, oral contraceptives, etc.
	• Primary hypothyroidism
	• Pregnancy
	• Breast sucking stimulation
	• Prolactinoma
100–200 ng/mL	• Prolactinoma if drug-induced cause is excluded
> 200 ng/mL	• Macro-prolactinoma
> 1000 ng/mL	• Macro-prolactinoma with cavernous sinus invasion
	• Giant (> 4 cm) prolactinoma

Pharmacotherapy with dopamine agonists (e.g., cabergoline) is preferred for PRL-producing tumors. However, because medications may cause the PitNET/pituitary adenoma to shrink or disappear on imaging or reduce the contrast between the PitNET/pituitary adenoma and the normal pituitary gland, it is advisable to perform an MRI prior to starting medication to confirm the presence of the PitNET/pituitary adenoma and to determine its size and signal intensity before treatment [68]. PRL-producing tumors may also show spherical-type amyloid deposition, and the amyloid deposited areas show nodular hypointensity on T2WI [91]. The presence of amyloid deposition should also be noted in this type of PRL-producing tumor, as dopamine agonists may not shrink the tumor [92]. If medication has already been started at the time of the initial MRI, the imaging report should be prepared with the possibility that the medication may have reduced the visualization of the tumor. Note that in the case of invasive lactotroph tumors, the shrinkage of the tumor due to medication may result in meningeal fistula or meningitis [93]. Pituitary gland size increases during pregnancy, and PitNET/pituitary adenoma size usually increases with discontinuation of dopaminergic agonists [68].

### Corticotroph PitNET/pituitary adenoma (ACTH-producing PitNET/pituitary adenoma)

Microadenomas comprise approximately half of all adrenocorticotropic hormone-producing tumors (Fig. 1) [68]. As there is no effective drug therapy and surgery is the only effective treatment, highly accurate imaging, including dynamic MRI, is necessary. If possible, imaging with a 3 T MRI is desirable [69]. In addition, adrenocorticotropin-producing PitNETs/pituitary adenomas tend to show faster contrast speed on dynamic MRI than other PitNETs/pituitary adenomas [68]. It may be useful to perform dynamic MRI with a high temporal resolution imaging technique such as golden-angle radial sparse parallel [94].

In cases where no PitNET/pituitary adenoma is found on MRI or when an ectopic pituitary PitNET/pituitary adenoma is suspected, venous sinus sampling of the inferior pyramidal and cavernous sinuses may be performed [95]. Venous sinus sampling is very accurate in diagnosing pituitary or ectopic lesions, but dynamic MRI is more accurate than venous sinus sampling in diagnosing the location (left or right localization) of the microadenoma [96]. Recently, 68 Ga adrenocorticotropin-releasing hormone (CRH) PET-CT, a molecular imaging technique targeting the CRH receptor expressed within corticotroph PitNETs/pituitary adenomas, has been developed and was reported to be capable of localizing and diagnosing corticotroph adenomas [97].

**Fig. 7:**
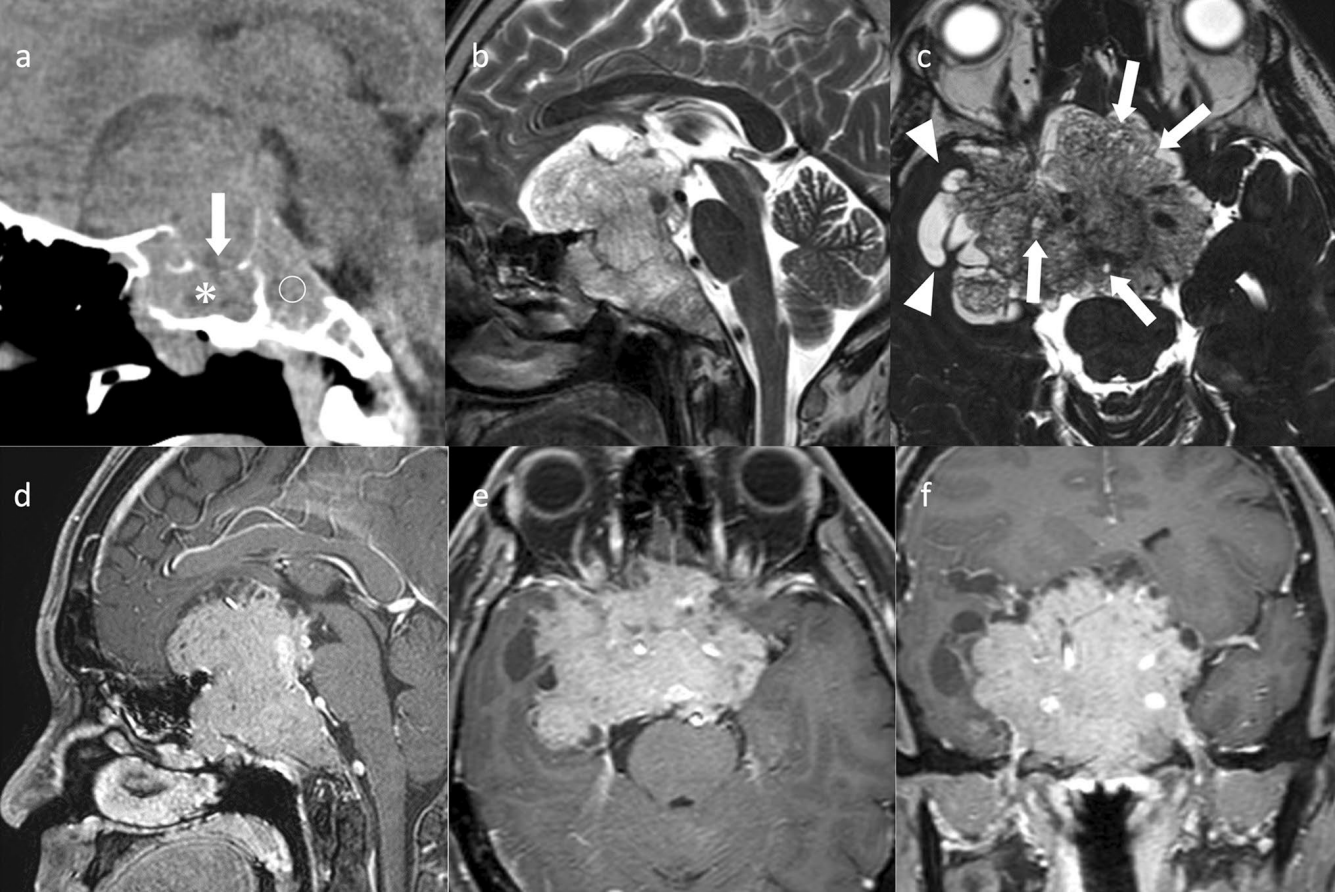
Silent corticotroph PitNET/pituitary adenoma. A male patient in his 40 s, with diplopia, disorientation, and cortisol levels within normal range. **a** Noncontrast CT shows destruction of the sella turcica (arrow) and invasion of the sphenoid sinus (asterisk) and the clivus (open circle). **b** Sagittal T2WI shows a mass with hyperintensity and iso-intensity areas. **c** Axial heavy T2WI shows small multiple cysts within the mass. Cysts around the mass likely represent trapped encysted pools of cerebrospinal fluid.** d–f** Contrast-enhanced T1WI shows a large mass centered in the sella turcica and invading the surrounding areas (the bilateral cavernous sinuses, and inferiorly, the sphenoid sinus and the clivus)

Silent corticotroph PitNET/pituitary adenoma usually present as a macroadenoma. Silent corticotroph adenomas are considered to have a more aggressive behavior than other clinically nonfunctioning PitNETs/pituitary adenomas with a higher rate of preoperative hypopituitarism, a higher prevalence of cavernous sinus invasion, and an earlier recurrence (Fig. 7) [98]. When multiple cysts are observed on T2WI in macroadenoma, silent corticotroph PitNET/pituitary adenoma is highly likely (sensitivity 58%, specificity 93%) [98]. It is theorized that the cysts are observed as a dissociated tissue with pseudopapillary dehiscences [99].

### Ectopic posterior lobe associated with macroadenoma

In macroadenomas, T1WI may show hyperintensity at the margins of the tumor (Fig. 4b) [100–102]. This hyperintensity area is believed to be an ectopic posterior lobe formed irreversibly at the distal portion of the pituitary stalk due to compression by a macroadenoma, because of the contrast enhancement in this hyperintensity area and the fact that the hyperintensity area does not recover in the normal position after surgery, but remains distal to the pituitary stalk [101]. The frequency of ectopic posterior lobe formation in PitNETs/pituitary adenomas is related to the size of the tumor, with cases of ectopic posterior lobe formation observed when the volume of the tumor exceeds 1 cc and a normally located (within the sella turcica) posterior lobe is not observed when the volume of the tumor exceeds 6 cc [101]. As an ectopic posterior lobe is capable of producing antidiuretic hormone, preoperative identification of the hyperintensity area of the ectopic posterior lobe may be useful in preventing permanent postoperative urinary retention [101]. Fat suppressed 3D T1-weighted volume isotropic turbo spin-echo acquisition (VISTA) has been reported to be more useful than conventional two-dimensional T1WI in the evaluation of ectopic posterior lobe cases [103].

### Pituitary apoplexy

Pituitary apoplexy is characterized by a sudden onset of headache, nausea, vomiting, visual impairment, disturbed consciousness, and hormonal dysfunction due to acute hemorrhage or infarction of the pituitary, usually with an existing PitNET/pituitary adenoma [104].

Pituitary apoplexy is a complication in 2–12% of cases of pituitary PitNET/pituitary adenoma, most of which are nonfunctioning PitNETs/pituitary adenomas; three of four pituitary tumors are initially diagnosed following onset of pituitary apoplexy [105]. While up to 25% of pituitary tumors exhibits hemorrhagic or necrotic regions, the absence of symptoms does not lead to a diagnosis of pituitary apoplexy [104]. Various factors that can induce pituitary apoplexy include hypertension, diabetes mellitus, pituitary function dynamic tests, administration of anticoagulants, bromocriptine, estrogens, and radiotherapy [106].

Many textbooks and review articles state that the primary cause of pituitary apoplexy is hemorrhage, but most are actually infarcts (hemorrhagic or non-hemorrhagic infarcts) and rarely are they simply hemorrhages without infarcts. In a study that confirmed this via surgery, the following rates were documented: hemorrhagic infarct, 47%; non-hemorrhagic infarct, 40%; and hemorrhage, 8% (surgery not performed, 5%) [107].

**Fig. 8:**
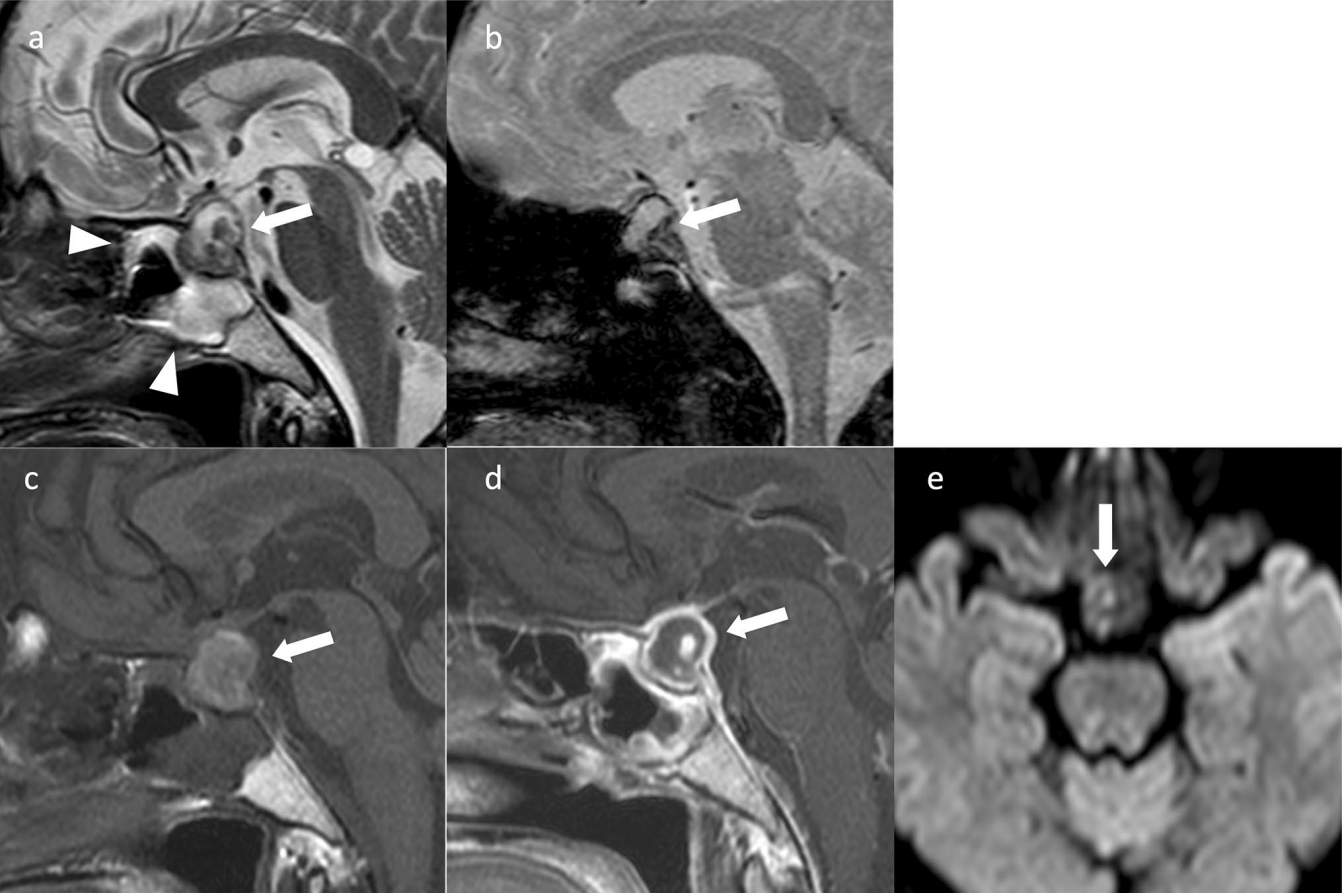
Pituitary apoplexy. A female patient in her 50 s, with headache, fever, and left ptosis. **a** Sagittal T2WI shows a mass in the sella turcica (arrow). The interior of the mass has a hyperintensity with some hypointensity regions. Mucosal thickening is observed in the sphenoid sinus (arrowhead). **b** Sagittal T2*WI reveals a hypointensity region in the mass, suggesting hemorrhage (arrow). **c** Sagittal T1WI shows a mildly hyperintensity mass (arrow). **d** Sagittal contrast-enhanced T1WI shows a ring-enhancing mass (arrow). **e** DWI shows iso-intensity (arrow). The patient underwent surgery via the transsphenoidal sinus approach, and hemorrhage and necrosis were observed

Various imaging findings are observed depending on the presence or absence of hemorrhage, including the timing [108]. If there is no hemorrhage in the infarct alone, the acute stage will show pituitary enlargement and hypointensity on T1WI, hyperintensity on T2WI, and hyperintensity on DWI [109]. If there is hemorrhage, changes suggestive of bleeding may be present, such as hyperintensity on T1WI, hypointensity on T2WI, and hypointensity on T2*-weighted image, depending on the timing [110]. Contrast-enhanced MRI does not reveal areas of infarction or hemorrhage, but rather reveal areas of residual PitNET/pituitary adenoma, showing a heterogeneous contrast enhancement (Fig. 8) [107]. Sphenoid sinus mucosal thickening is observed in approximately 80% of cases during the acute (≤ 1 week) stage of pituitary apoplexy [111]. Sphenoid sinus mucosal thickening may correlate with higher grades of pituitary apoplexy and worse neurological/endocrinological outcomes [112]. In addition, thickening of the mucosa of the sphenoid sinus, if present, is often treated surgically [113].

Sheehan’s syndrome is a condition in which infarction and necrosis of the pituitary gland occur after a major hemorrhage during parturition [114]. Sheehan syndrome, which is a pituitary stroke occurring independently of a PitNET/pituitary adenoma, shows a pituitary enlargement, with hypointensity on T1WI, hyperintensity on T2WI and rim-like enhancement of the pituitary gland on contrast-enhanced T1WI [115].

In women in late pregnancy and the postpartum period, the anterior pituitary gland may be enlarged and show hyperintensity on T1WI [116, 117]. It is important not to misconstrue this for pituitary hemorrhage or PitNET/pituitary adenoma with hemorrhage.

### Ectopic PitNET/pituitary adenoma (Fig. 9)

**Fig. 9:**
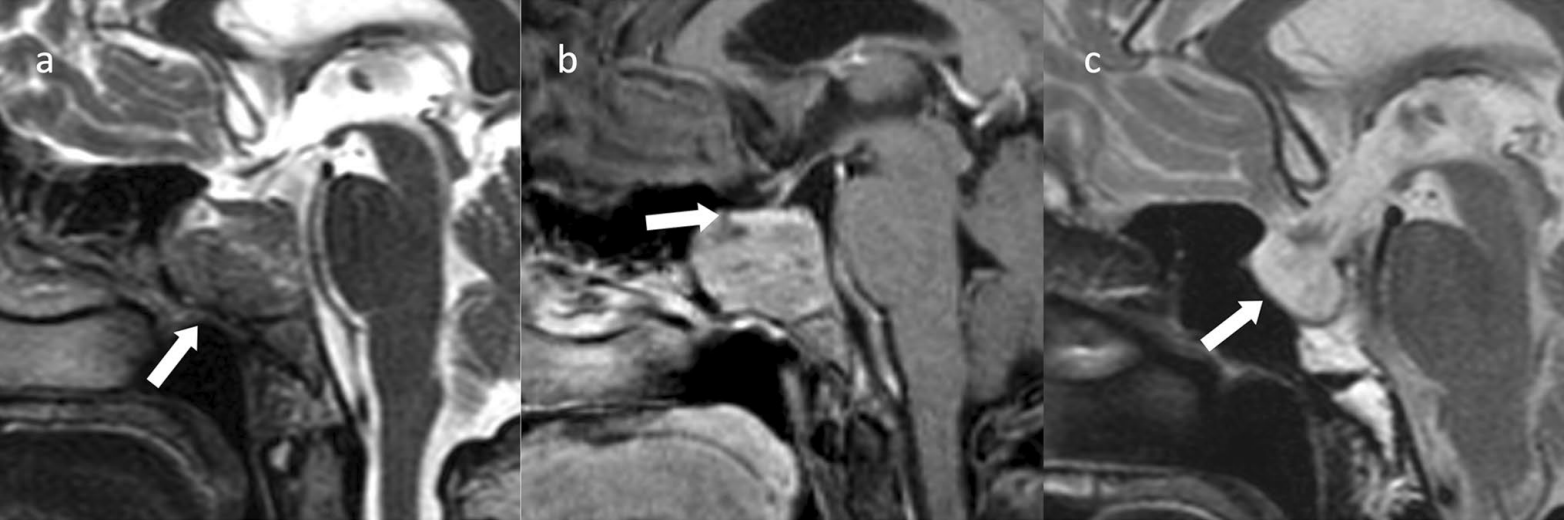
Ectopic PitNET/pituitary adenoma (prolactinoma). A female patient in her 40 s, with an incidentally discovered tumor. The PRL level was at 4900 ng/mL which is markedly elevated. a Sagittal T2WI shows a mass in the base of the sella turcica (arrow). b Contrast-enhanced T1WI shows a pituitary gland contrasted flat above the mass (arrow). The contrast enhancement of the mass is weak compared to the pituitary gland. c Sagittal T2WI after 2 years of oral cabergoline shows tumor shrinkage and empty sella (arrow). PRL was 91 ng/mL and is nearly normal

An ectopic PitNET/pituitary adenoma is a PitNET/pituitary adenoma discovered outside the sella turcica that shows no association with the normal pituitary gland [118]. It is believed to arise from ectopic pituitary tissue left behind when the pouch of Rathke migrates from the primordial oral cavity [118]. Ectopic PitNETs arise in the sphenoid sinus or upper nasopharynx and less frequently in the ethmoid sinus and nasal bridge [119]. PitNETs/pituitary adenomas may be observed in the suprasellar region, but most are originated from the pars tuberalis of the pituitary stalk, and true ectopic PitNETs/pituitary adenomas without continuity with pars tuberalis are rare [120].

Approximately 58% of patients present with symptoms of hormonal excess, such as Cushing’s syndrome, acromegaly, or hyperparathyroidism [118]. If symptoms of hormonal excess are present and there is no PitNET/pituitary adenoma within the sella turcica, searching for an ectopic PitNET/pituitary adenoma in the sphenoid sinus or elsewhere becomes necessary [121].

### Postoperative imaging diagnosis of PitNET/pituitary adenoma

Postoperative imaging should be performed to check for the status and presence or absence of residual PitNET/pituitary adenoma, the release of pressure on surrounding structures, and the presence of bleeding. In PitNET/pituitary adenoma surgery, fillers are often inserted into the extraction cavity. Fillers include subcutaneous fat, muscle, gelatin sponge, gelatin-containing human thrombin, and absorbent local hemostatic agents, and the signal strength of the gelatin sponge varies depending on the absorbed blood component. Contrast-enhanced MRI is useful in differentiating between filling, residual PitNET/pituitary adenoma, normal pituitary, and inflammatory tissue [69]. Dynamic MRI is also useful to differentiate postoperative changes and residual PitNET/pituitary adenoma. Residual tumors are reported to exhibit a nodular contrast area on dynamic MRI [122], and no residual tumors have been reported to be present when the contrast area is visible only at the margins [123].

### Fluorodeoxyglucose (FDG)-PET accumulation

**Fig. 10:**
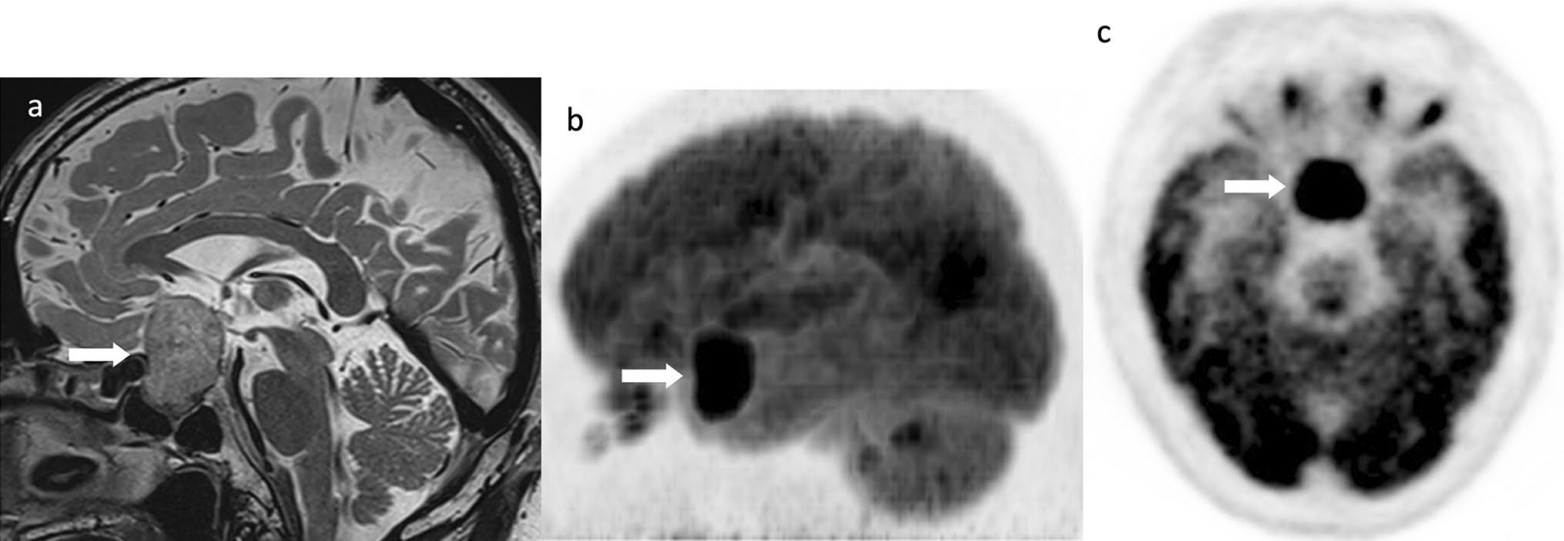
Fluorodeoxyglucose (FDG)-positron emission tomography accumulation in a PitNET/pituitary adenoma. A male patient in his 50 s, with a nonfunctioning PitNET/pituitary adenoma. **a **Sagittal T2WI revealed a mass within the sella turcica to the suprasellar region (arrow). **b**, **c** Marked FDG accumulation in the mass (arrow) (SUVmax = 20.1). Courtesy of Dr. Yuji Nakamoto

PitNETs/pituitary adenomas are highly concentrated on FDG-PET and may be detected incidentally during PET examinations or in the search for metastases of malignant tumors (Fig. 10). They may be functional or nonfunctioning PitNETs/pituitary adenomas, macroadenomas, or microadenomas. PET-MRI may be useful in detecting microadenomas because FDG also accumulates in microadenomas [124]. Although this used to be exceptional in showing hyperaccumulation on FDG-PET in PitNETs/pituitary adenomas, which are benign tumors, this is no longer the case as they are now classified as malignant tumors in the recent revision of the WHO classification. When high FDG accumulation is observed near the sella turcica, PitNET/pituitary adenoma should be excluded first through MRI and/or endocrinological examination, rather than suspect other malignancy.

## Conclusions

The PitNET/pituitary adenoma is the most common pathology of the pituitary region. In proposing a name change from pituitary adenoma to PitNET, the 5th edition of the WHO classification made an important and significant change in the fundamental concept of the disease. Imaging has a major role in the diagnosis and evaluation of progression, and it is important to know the appropriate imaging methods and strategies for PitNET/pituitary adenoma based on knowledge of size and clinical presentation. In addition, radiologists should consider the changes in the latest WHO classification in their diagnostic imaging reports.
