# Correction to: Imaging of pituitary tumors: an update with the 5th WHO Classifications—part 1. Pituitary neuroendocrine tumor (PitNET)/pituitary adenoma

**DOI:** 10.1007/s11604-023-01414-1

**Published:** 2023-03-24

**Authors:** Taro Tsukamoto, Yukio Miki

**Affiliations:** Department of Diagnostic and Interventional Radiology, Graduate School of Medicine, Osaka Metropolitan University, 1-4-3 Asahi-Machi, Abeno-Ku, Osaka, 545-8585 Japan

**Correction to: Japanese Journal of Radiology (2023)** 10.1007/s11604-023-01400-7

In the original publication, part b in caption of Fig. 5 should read as:

Sagittal T1WI shows hyperintensity anteriorly and mildly hypointensity posteriorly.

Reference 2 should read as:

WHO Classification of Tumours Editorial Board. Endocrine and Neuroendocrine tumours [Internet].

Lyon (France): International Agency for Research on Cancer; 2022 [cited 2022 Nov 1]. (WHO classification of tumours series, 5th ed.; vol. 10). https://tumourclassification.iarc.who.int/chapters/53.

The original publication has been corrected.

